# Investigation on LiBH_4_-CaH_2_ composite and its potential for thermal energy storage

**DOI:** 10.1038/srep41754

**Published:** 2017-01-31

**Authors:** Yang Li, Ping Li, Xuanhui Qu

**Affiliations:** 1 State Key Laboratory for Advanced Metals and Materials, Institute for Advanced Materials and Technology, University of Science and Technology Beijing, Beijing 100083, China

## Abstract

The LiBH_4_/CaH_2_ composite are firstly studied as Concentrating Solar Power Thermal Storage Material. The LiBH_4_/CaH_2_ composite according to the stoichiometric ratio are synthesized by high-energy ball milling method. The kinetics, thermodynamics and cycling stability of LiBH_4_/CaH_2_ composite are investigated by XRD (X-ray diffraction), DSC (Differential scanning calorimeter) and TEM (Transmission electron microscope). The reaction enthalpy of LiBH_4_/CaH_2_ composite is almost 60 kJ/mol H_2_ and equilibrium pressure is 0.482 MPa at 450 °C. The thermal storage density of LiBH_4_/CaH_2_ composite is 3504.6 kJ/kg. XRD results show that the main phase after dehydrogenation is LiH and CaB_6_. The existence of TiCl_3_ and NbF_5_ can effectively enhance the cycling perfomance of LiBH_4_/CaH_2_ composite, with 6–7 wt% hydrogen capacity after 10 cycles. The high thermal storage density, high working temperature and low equilibrium pressure make LiBH_4_/CaH_2_ composite a potential thermal storage material.

Solar energy is the most plentiful renewable and clean alternative to fossil fuels^1^. International Energy Agency (IEA) points out that solar energy will make up 22 percent of the global electricity, and it is possible that solar photovoltaics (PV) and concentrating solar thermal (CST) power technology will play roughly equal, but complementary roles by 2050^2^. The CST power technology can store energy as heat that can be assessed on demand to generate electricity when PV technology is inefficient, such as at night or during rainy days.

As thermal storage (TS) material is the key element in the CST, improving the energy storage density and working temperature have great value on power generation efficiency and cutting back on the cost. There are three basic methods of thermal storage. Considering the condition of CST plants, sensible heat storage with molten salt is of low-efficiency and can be corrosive sometimes and latent heat storage using NaNO_3_ is of high flammability and reactivity and is uncertain over its longevity. Thermochemical heat storage materials have quite high energy storage density, in which hydrides’ exceed 1700–4000 kJ kg^−1^ (10~30 times more than molten salts’ energy storage density and 4~10 times more than phase change materials’ energy storage density)^3^,^4^. The characteristics of common and potential thermal storage materials are listed in Table 1.

Among all hydrides, complex hydrides such as LiBH_4_, NaAlH_4_ and NaBH_4_ possess a quite high forming enthalpy due to the transition to an ionic or covalent compound of metals upon hydrogen absorption^9^ It seems that complex hydrides are promising thermal storage materials in CST plants.

In fact, LiBH_4_ is mostly researched as a hydrogen storage material due to the second highest hydrogen content (18.4 wt.%) of all alanates and boranates^10^,^11^,^12^,^13^. The thermal hydrogen desorption of pure LiBH_4_ starts at ~320 °C and proceeds mainly in the temperature region 400–600 °C, which is in accordance with the working temperature of thermal storage material in CST plant.


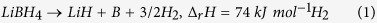


However, the sluggish kinetics and poor reversibility of LiBH_4_ are the problems that limit its use in hydrogen storage^11^,^12^,^13^. The destabilization was proposed to change the reaction process by adding a reactive additive^14^. Thus, many new systems have been proposed based on DFT calculation of reaction enthalpies in multi-component systems^15^,^16^. Among all systems, LiBH_4_-CaH_2_ is one of promising composites that are suitable for thermal storage due to its onset temperature. And this reaction can produce around 11.7 wt% hydrogen. But Yang reported that LiBH_4_-CaH_2_ composite is irreversible under the condition tested (350 °C, 150 bar)^17^. The sluggish kinetics in LiBH_4_-CaH_2_ composite is another problem that need to be solved^18^.


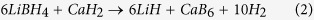


Additives, such as TiCl_3_^19^,^20^,^21^,^22^,^23^, V_2_O_5_^19^, TiF_3_^19^,^20^, TiO_2_^19^,^20^, LiNH_2_^19^, NbF_5_^24^,^25^, NbCl_5_^24^ were investigated their effects on the kinetics and cycling performance of LiBH_4_-CaH_2_ composite. Results show that TiCl_3_ additive is very effective in lowering activation energy of dehydrogenation and enhancing reversibility (about 9 wt% hydrogen reversibly)^19^,^20^,^21^,^22^,^23^. Besides, Lim reported that LiBH_4_-CaH_2_ composite with NbF_5_ maintains a reversible hydrogen storage capacity of about 6 wt% at 450 °C^25^.

But the enthalpies of pure LiBH_4_-CaH_2_ composite and LiBH_4_-CaH_2_ composite with additives have not been reported exactly. Pinkerton^21^ reported the estimated enthalpy of reaction 2 at 400 °C is ΔH = 59.2 kJ/mol H_2_. Lim^24^ reported that the reaction enthalpy of LiBH_4_-CaH_2_-0.2NbF_5_ composite is estimated to be 56.5 kJ/mol H_2_ at 305 °C. But according to HSC thermochemical database^21^, the enthalpy is 66.2 kJ/mol H_2_ at room temperature.

In this research, the enthalpy ΔH and equilibrium pressure of desorption according to reaction 2 are determined by PCT (pressure, concentration, and temperature) measurements. Only LiBH_4_-CaH_2_ without catalysts was measured due to the fact that influence the thermodynamics of the mixture as Ti-doped NaAlH_4_^12^. The additives such as TiCl_3_ and NbF_5_ are investigated their effect on kinetics and cycling performance of LiBH_4_-CaH_2_ composite.

## Experimental Details

LiBH_4_ (≥95% pure), purchased from Acros Organics, CaH_2_ (≥98% pure) and NbF_5_ ( ≥ 99% pure), purchased from Alfa Aesar, and TiCl_3_ (≥95% pure), synthesized by the reaction of titanium tetrachloride with metallic titanium in molten CaCl_2_ and the enrichment process with HCl gas^26^, were utilized directly without any further purification. The mole ratio of LiBH_4_-CaH_2_ composite according to reaction 2 is 6:1. The pure LiBH_4_-CaH_2_ composite and LiBH_4_-CaH_2_ composite doped with different additives (1 mol% TiCl_3_, and 5 wt% NbF_5_) was ball-milled under argon atmosphere by using a QM-2B high energy mill (Nanjing NanDa Instrument Plant) at a rotating speed of 1200 rpm for 1 h. Two kinds of stainless steel balls with 4 mm and 8 mm diameters were added with a ball-to powder weight ratio of 12.5:1. Typically, 4 g mixture was sealed in the stainless steel vessel within a high purity argon atmosphere during milling. To avoid excess heating of the stainless steel vessel, there were 10 min intervals between each 5 min milling process.

The isothermal desorption was measured by using the Sieverts-type pressure-composition-temperature (P-C-T) apparatus (General Research Institute for Nonferrous Metals, China). The maximum pressure, maximum vacuum degree and maximum temperature of this apparatus is 10 MPa, 10^−1^ Pa and 800 °C, respectively. Typically, 60–100 mg sample was loaded into the vessel, and then heated up to 450 °C under 0.1 MPa hydrogen atmosphere. Following the dehydrogenation, the samples were subjected to rehydrogenation studies at 450 °C under 8 MPa hydrogen pressure for 16 h. It should be noted that the additional content was not taken into consideration when calculating the released hydrogen. The PCI (Pressure-composition isotherms) curves were measured at 405 °C, 420 °C, 435 °C, 450 °C and 465 °C, respectively.

The phase structure of the samples after milling and dehydrogenation was examined by an MXP21VAHF X-ray Diffractometer (XRD with Cu Kα radiation, 40 kV, 300 mA), with the 2θ angle ranged from 10° to 90° with a scanning rate of 10° min^−1^. X-ray photoelectron spectroscopy (XPS) was performed with the PHI-5300 spectrometer. The morphology and phase constitution of all samples after ball milling and desorption were observed by and transmission electron microscopy (Tecnai G2 F30 S-TWIN, FEI, USA). Simultaneous differential scanning calorimetry (DSC) and Thermogravimetric Analysis (TGA) experiments were conducted under 50 mL min^−1^ argon flow in a NETZSCH STA 449F3 Jupiter instrument between 50 °C and 500 °C with a heating rate of 5 °C min^−1^. The samples were transferred to Al_2_O_3_ crucibles under argon atmosphere for the DSC-TGA measurements.

All samples handling was performed under strictly inert conditions (≥99.99% Ar atmosphere) in the glove box (Mikrouna, Super-750) equipped with oxygen/humidity sensors and recirculation system to avoid oxidation and moisture. Oxygen and H_2_O levels were kept below 0.1 ppm.

## Results and Discussion

### XPS characterization

**Figure 1:**
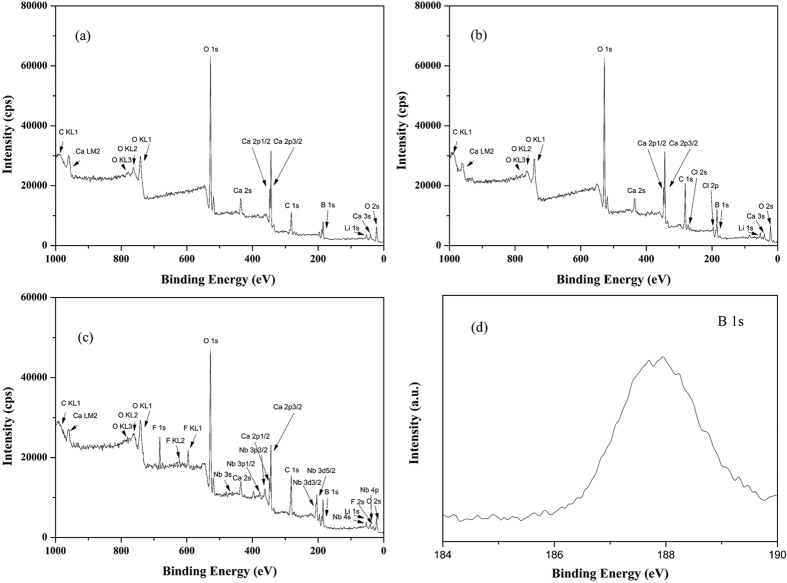
XPS scan spectra of three LiBH_4_-CaH_2_ composites after ball milling: (**a**) pure composite (**b**) 1 mol% TiCl_3_ addition (**c**) 5 wt% NbF_5_ addition (**d**) B 1 s in pure composite.

The XPS results of three LiBH_4_-CaH_2_ composites after milling are presented in Fig. 1(a–c), which confirms the existence of element Li, B and Ca in both composites. Element Nb, F and Cl are identified in the catalyst-doped composite, while Ti are not discovered due to the low amount addition. The XRD results are presented in Fig. S1. There are only two obvious peaks in both composites, which are characterized as CaH_2_. It can be inferred that the structure of LiBH_4_ after milling becomes amorphous. No peaks of LiBH_4_, TiCl_3_ or NbF_5_ are detected. The XPS narrow spectra of ball-milled LiBH_4_-CaH_2_ composite are showed in Fig. 1(d). The photo-emission spectrum of B 1 s at 187.8 eV corresponds to LiBH_4_, while the existence of LiCl and NbF_5_ are also convinced in Fig. S2. XPS results testify that LiBH_4_ and TiCl_3_ react during ball milling^22^.

### Investigation on energy storage density

The energy storage density is the most significant factor when evaluating a material is suitable for TS. The energy storage density to weight is related with the reaction enthalpy and molar mass. For dehydrogenation reaction of hydrides, the Van’t hoff equation and DSC integration can be used to calculate the reaction enthalpy. Besides high energy storage density, low equilibrium pressure and high working temperature are also two important factors in selecting hydrides for TS.

### PCI curves and Van’t Hoff Calculation

**Figure 2:**
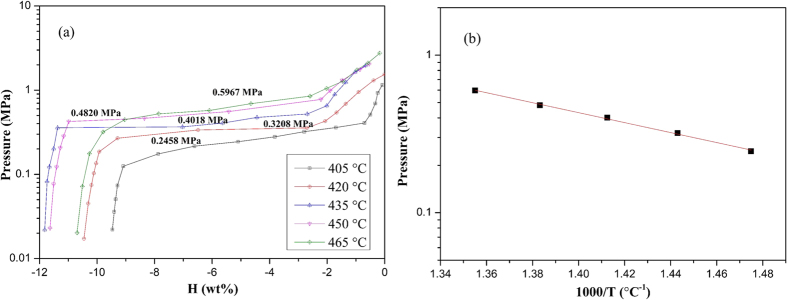
(**a**) Dehydrogenation PCI curves, (**b**) Dehydrogenation van’t hoff curves of pure LiBH_4_-CaH_2_ composite.

The dehydrogenation PCI curves of pure LiBH_4_-CaH_2_ composite are shown in Fig. 2(a). Due to the sluggish kinetics, the pressure value in each platform can only be read after 4-hours waiting. Even so, the platform inclination is quite a lot, especially in low temperature condition. Considering the platform is a slope to some extent, the equilibrium pressures from 405 °C to 465 °C are calculated as the mean of pressure values in the platform. The equilibrium pressures of pure LiBH_4_-CaH_2_ composite from 405 °C to 465 °C are 0.2458 MPa, 0.3208 MPa, 0.4018 MPa, 0.4820 MPa and 0.5967 MPa, respectively. They are lower than the equilibrium pressures of reported thermal storage metal hydrides, such as MgH_2_, Mg_2_FeH_6_ and Ce_2_Mg_17_H_x_^4^,^6^,^8^. What’s more, the low equilibrium pressure makes LiBH_4_-CaH_2_ composite possible to be operated at higher temperature. The higher working temperature can increase overall solar to electricity conversion efficiency and reduce the cost in CST plants^27^. The dehydrogenation capacity of pure LiBH_4_-CaH_2_ composite is mostly ranging from 10.5 wt% to 11.6 wt%, which is close to their theoretical value. The sluggish kinetics resulting from the relatively low temperature (405 °C) may account for the lower capacity (9.5 wt%). Only LiBH_4_-CaH_2_ without catalysts was measured due to the fact that influence the thermodynamics of the mixture as Ti-doped NaAlH_4_^12^. Liu^22^ reported that LiCl forms during ball milling of 6LiBH_4_/CaH_2_/xTiCl_3_. LiF and CaF_2_ are observed after the ball milling reaction of NbF_5_ and LiBH_4_ or CaH_2_^24^. Thermodynamics of pure LiBH_4_-CaH_2_ composite might have changed due to the formation of LiCl or LiF and CaF_2_.

A plot of ln P against 1000/T in Fig. 2(b) results in a nearly straight line. Calculation of Δ*H* = *R* · (ln*P*_2_ − ln*P*_1_)/(1/*T*_2_ − 1/*T*_1_) from ln P and 1/T values at 405 °C and 465 °C provides a ΔH of 60.555 kJ mol^−1^ H_2_. According to the reaction 2, a thermal storage density value of 3504.6 kJ kg^−1^ is calculated. It shows a superior capacity to sensible and latent thermal storage materials, even to thermochemical thermal storage materials shown in Table 1.

### DSC Calculation

**Figure 3:**
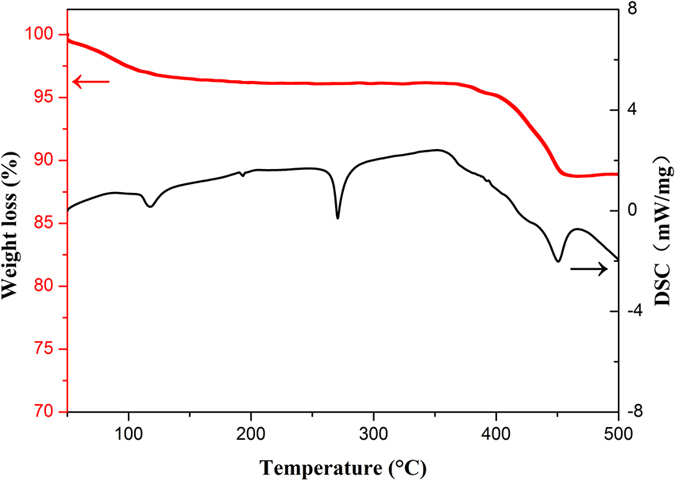
DSC and TGA curves of pure LiBH_4_-CaH_2_ composite.

The DSC and TGA curves of pure LiBH_4_-CaH_2_ composite are shown in Fig. 3. There are mainly three endothermic peaks during the heating process. The endothermic effect at 108–112 °C is reversible and corresponds to polymorphic transformation of LiBH_4_. The second peak at 268–286 °C corresponds to the fusion of LiBH_4_. The third peak corresponds to the dehydrogenation behavior of LiBH_4_. The onset temperature is 392 °C and the peak temperature is 446 °C. According to TGA results, dehydrogenation reaction ends at 497 °C. The integration of DSC on temperature from 392 °C to 497 °C is calculated as enthalpy of reaction 2, with a value of 60.706 kJ mol^−1^ H_2_.

The DSC and TGA curves of LiBH_4_-CaH_2_ composites with TiCl_3_ and NbF_5_ addition are shown in Fig. S3. There are both three endothermic peaks in these two composites. NbF_5_ addition shows a more remarkable influence on the decrease of onset temperature than TiCl_3_. The onset temperature, dehydrogenation reaction enthalpy and thermal storage density of three composites and other potential TS system are shown in Table 2. The TiCl_3_ doped composite and NbF_5_ doped composite shows similar reaction enthalpy as the pure LiBH_4_-CaH_2_ composite. It can be speculated that catalyst additions in LiBH_4_-CaH_2_ composite do not have a remarkable influence on the dehydrogenation reaction enthalpy. The TS density of pure composite is the highest (3511.45 kJ kg^−1^), while TiCl_3_ and NbF_5_ doped composites possess nearly 3300 kJ kg^−1^ TS density, with a little reduction. The DSC calculation results are in accordance with the Van’t hoff calculation results. Comparing with actual TS density of MgH_2_ (2147 kJ kg^−1)^^28^,^29^, LiBH_4_-CaH_2_ composites shows a clear superiority.

### Investigation on kinetics

**Figure 4:**
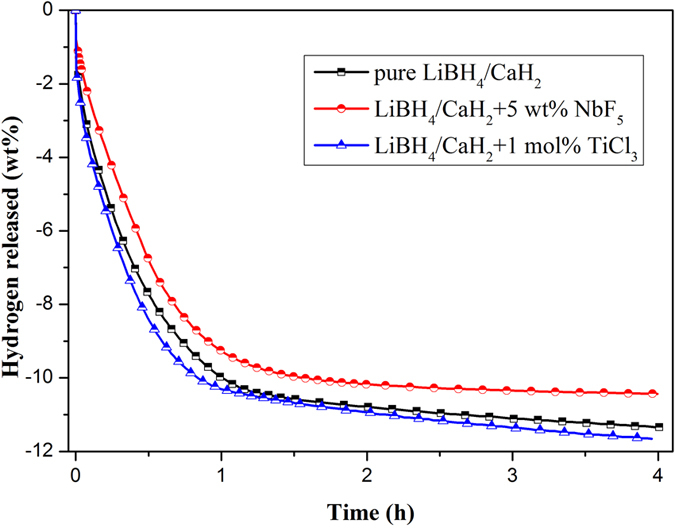
Isothermal dehydrogenation curves of different LiBH_4_-CaH_2_ composites under 450 °C and H_2_ atmosphere (0.1 MPa).

The Fig. 4 shows the desorption behavior of three LiBH_4_-CaH_2_ composites. The addition of TiCl_3_ significantly improves the dehydrogenation kinetics of LiBH_4_-CaH_2_ composite, while NbF_5_ influence it in an opposite way. Both composites can release 9–10 wt% hydrogen in an hour. After 4 hours, the pure composite and TiCl_3_ doped composite shows a nearly theoretical hydrogen capacity (11.7 wt%), while NbF_5_ doped composite only desorbs around 10 wt% hydrogen. TiCl_3_ shows a remarkable impact on improving the desorption kinetics and maintaining the hydrogen capacity. Liu^22^ reported that LiCl formed through replacement reaction between LiBH_4_ and TiCl_3_ during ball milling can be incorporated into LiBH_4_ to form solid solution LiBH_4_·LiCl. It favorably changes viscosity, preserving the nano-sized phase arrangement formed after milling, leading to fast kinetics.

**Figure 5:**
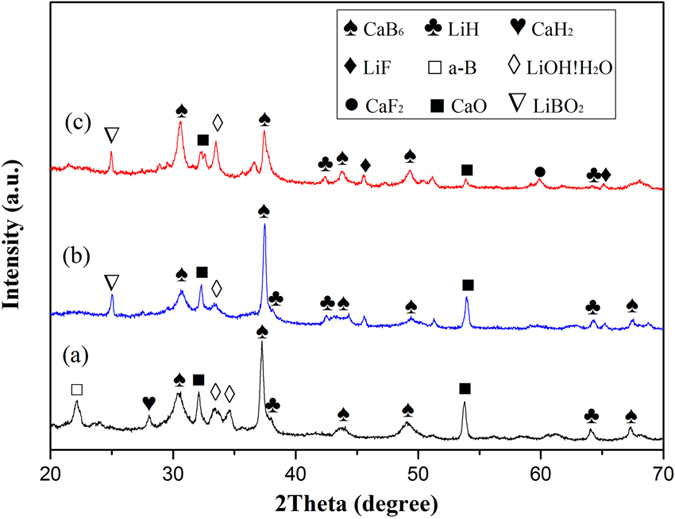
XRD patern of three LiBH_4_-CaH_2_ composites after desorption: (**a**) pure composite (**b**) 1 mol% TiCl_3_ addition (**c**) 5 wt% NbF_5_ addition.

The XRD results of three LiBH_4_-CaH_2_ composites after dehydrogenation are shown in Fig. 5. The main phase of both composites is LiH and CaB_6_, which is in accordance with reaction 2. The existence of phase CaO, LiOH!H_2_O and LiBO_2_ is due to the oxidation during the experiments. The remaining CaH_2_ and B are identified in the pure composite after dehydrogenation. B is the product of LiBH_4_ after dehydrogenation (shown in reaction 1), which also explains why a little CaH_2_ remains. The LiF and CaF_2_ phase are detected in the NbF_5_ doped composite, while no peaks of chlorides are identified in the TiCl_3_ doped composite.

### Investigation on reversibility and cycling stability performance

**Figure 6:**
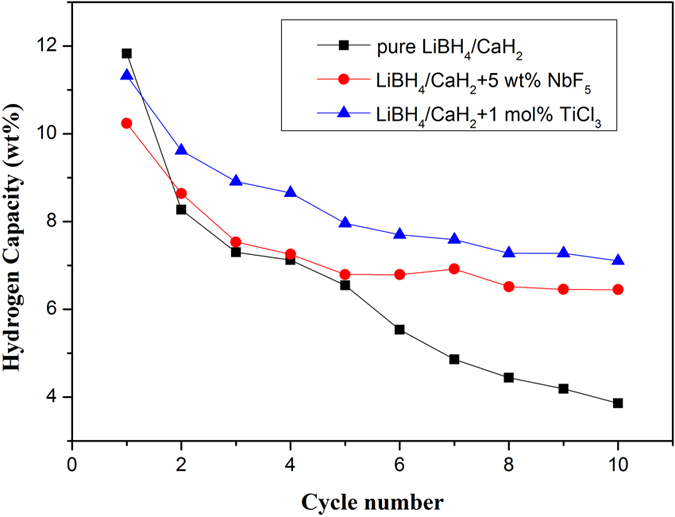
Cycling curves of three LiBH_4_-CaH_2_ composites.

A test over 10 cycles was performed under very severe cycling conditions (desorption: 450 °C, 0.1 Mpa,4 h; absorption: 450 °C, 8 MPa, 16 h). The results are shown in Fig. 6. The initial hydrogen capacity of the pure composite and TiCl_3_ doped composite shows a nearly theoretical hydrogen capacity (11.7 wt%), while NbF_5_ doped composite only desorbs around 10 wt% hydrogen. The hydrogen capacity of both composites declines during cycling. It is worth mentioning that TiCl_3_ doped composite can reversibly store 9 wt% hydrogen during first three cycles. After 10 cycles, the remaining hydrogen capacity of pure composite, NbF_5_ doped composite and TiCl_3_ doped composite is 3.8 wt%, 6.4 wt% and 7.1 wt%, respectively. TiCl_3_ and NbF_5_ seems effectively raise the cycling stability performance of LiBH_4_-CaH_2_ composite.

**Figure 7:**
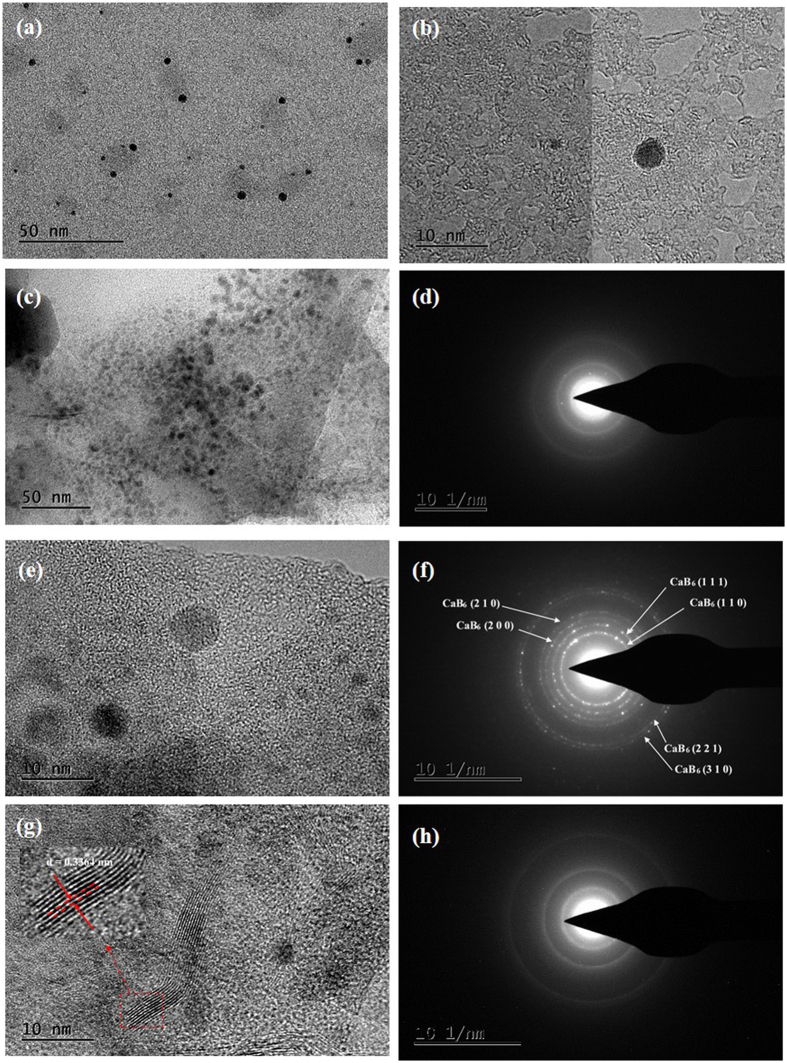
TEM and SAED images: (**a**–**d**) pure LiBH_4_-CaH_2_ composite after 10 cycles; (**e**,**f**) TiCl_3_ doped composite after 10 cycles; (**g**,**h**) of NbF_5_ doped composite after 10 cycles.

The TEM images of pure LiBH_4_-CaH_2_ composite after 10 cycles are shown in Fig. 7(a–d). The main phases are small particles with a diameter of 3–6 nm, separately scattering. Particle aggregation shown in Fig. 7(c), which may result from the sintering, is also found. The diffraction ring in Fig. 7(d) is very obvious, indicating that amorphous structure is formed. The particle aggregation and amorphous structure of products accounts for the dramatic loss of hydrogen capacity of pure LiBH_4_-CaH_2_ composite during cycling. TEM images of TiCl_3_ doped composite and NbF_5_ doped composite after 10 cycles are shown in Fig. 7(e,f,g and h). The small particles with a diameter of 3–6 nm are both observed. However, the results of electron diffraction indicate that the TiCl_3_ doped composite after 10 cycles is crystal structure, while NbF_5_ doped composite after 10 cycles is amorphous structure. By analyzing the diffraction ring diameter, the crystal structure is assumed to be CaB_6_. The amorphous structure of B is not good for the reverse reaction to produce LiBH_4_, while the crystal structure of CaB_6_ is in favor of the reverse reaction^29^,^31^,^32^. This explains why TiCl_3_ plays a more effective role in raising the cycling stability performance of LiBH_4_-CaH_2_ composite than NbF_5_. Moreover, it is noteworthy that a graphene-like lamellar structure are found in NbF_5_ doped composite after 10 cycles. The value of interlamellar spacing (d) is 0.3364 nm, which is corresponding to NbF_5_. But the lamellar structure of NbF_5_ is never reported. Thus, the d value of NbB_2_ is 0.3321 nm, which is close to the 0.3364 nm. Minella reported that NbB_2_ nanoparticles was observed after milling or upon sorption reactions of Nb-based Ca(BH_4_)_2_ doped composites^33^. It is reasonable to be assume that a small amount of NbB_2_ can also be formed in Nb-based LiBH_4_-CaH_2_ doped composites. It needs more research work to identify this graphene-like lamellar structure in NbF_5_ doped composite.

## Conclusion

The reaction enthalpy of LiBH_4_/CaH_2_ composite is almost 60 kJ/mol H_2_ and equilibrium pressure is 0.482 MPa at 450 °C. The thermal storage density of LiBH_4_/CaH_2_ composite is 3504.6 kJ/kg. XRD results show that the main phase after dehydrogenation is LiH and CaB_6_. The exsience of TiCl_3_ and NbF_5_ can effectively enhance the cycling perfomance of LiBH_4_/CaH_2_ composite, with 6–7 wt% hydrogen capacity after 10 cycles. The high thermal sotrage density, high working temperature and low equilibrium pressure make LiBH_4_/CaH_2_ composite a potential thermal storage material.

Although the high price of starting materials, such as LiBH_4_, will limit its usage, the LiBH_4_/CaH_2_ composite could serve as the additives for Magnesium-based alloys in TS. The research will be continued on the pair study of LiBH_4_/CaH_2_ composite with another metal hydride working at lower temperature.

## Additional Information

**How to cite this article**: Li, Y. *et al*. Investigation on LiBH_4_-CaH_2_ composite and its potential for thermal energy storage. *Sci. Rep.* **7**, 41754; doi: 10.1038/srep41754 (2017).

**Publisher's note:** Springer Nature remains neutral with regard to jurisdictional claims in published maps and institutional affiliations.

## Supplementary Material

Supporting Information

## Figures and Tables

**Table 1:** Energy stored per mass of different storage materials^5^,^6^,^7^,^8^.

Material	Sensible		Latent		Thermochemical	
	Rock	Concrete	Paraffin wax	NaNO_3_	CaCl_2_·H_2_O	MgH_2_
Specific heat capacity (kJ kg^−1^)	0.9	1.13	—	—	3.06	—
Latent heat of fusion (kJ kg^−1^)	—	—	174.4	172	—	—
Reaction enthalpy (kJ kg^−1^)	—	—	—	—	433.6	2860

**Table 2:** TS properties of three LiBH_4_-CaH_2_ composites and other potential TS composites^4^,^6^,^8^,^9^,^30^.

	H_2_ content (wt%)	Enthalpy (kJ mol^−1^ H_2_)	TS density (kJ kg^−1^)	Equilibrium temperature at 1 bar [°C]
MgH_2_	7.6	74.4	2860	280
NaMgH_3_^*^	4.0	86	1700	380
Mg_2_FeH_6_	5.5	77.4	2106.5	320
Mg_2_NiH_6_	3.6	62	1116	250
CaH_2_	4.8	186.2	4422.8	950
Ce_2_Mg_17_H_x_	5.0	75.5	1926.3	310
LiBH_4_-CaH_2_	11.7	60.706	3511.45	350
LiBH_4_-CaH_2_-1 mol% TiCl_3_	11.6	59.354	3316.64	—
LiBH_4_-CaH_2_-5 wt% NbF_5_	10.4	60.011	3307.71	—

*Calculated for the reaction 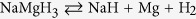.
