# Supplementary material for: Investigation on LiBH4-CaH2 composite and its potential for thermal energy storage
Source: Sci Rep. 2017 Jan 31;7:41754. doi: 10.1038/srep41754 (PMC5282489; doi:10.1038/srep41754)
Supplement: Supporting Information [file srep41754-s1.pdf]

Investigation on $LiBH_4$-CaH_2 composite and its potential for thermal energy storage

Yang Li , Ping Li *, Xuanhui Qu

State Key Laboratory for Advanced Metals and Materials, Institute for Advanced Materials and Technology, University of Science and Technology Beijing, Beijing 100083, China

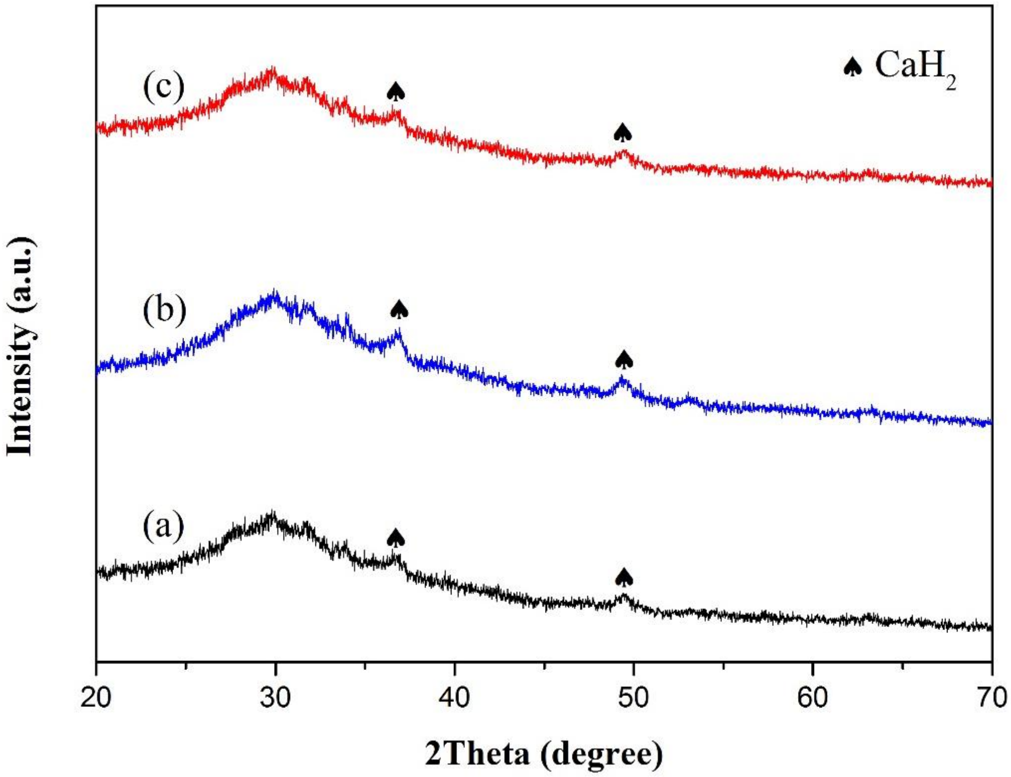

Figure S1 XRD pattern of three $LiBH_4$-CaH_2 composites:
(a) pure composite (b) 1 mol%$TiCl_3$ doped (c) 5 wt% NbF_5 doped

Fig. S2 shows the XPS scan spectra of ball-milled $LiBH_4$-CaH_2 composite with $TiCl_3$ and NbF_5 addition. Fig. S2(a) shows the photo-emission spectrum of Cl 2p at 199.1 eV, corresponding to LiCl. Fig. S2(b) shows the photoemission spectrum of Nb 3d at 207 eV and 209.8 eV, corresponding to NbF_5. The XPS results convince the existence of LiCl and NbF_5.

Fig. S3 exhibits the DSC and TGA curves of $LiBH_4$-CaH_2 composites with catalysts additions.

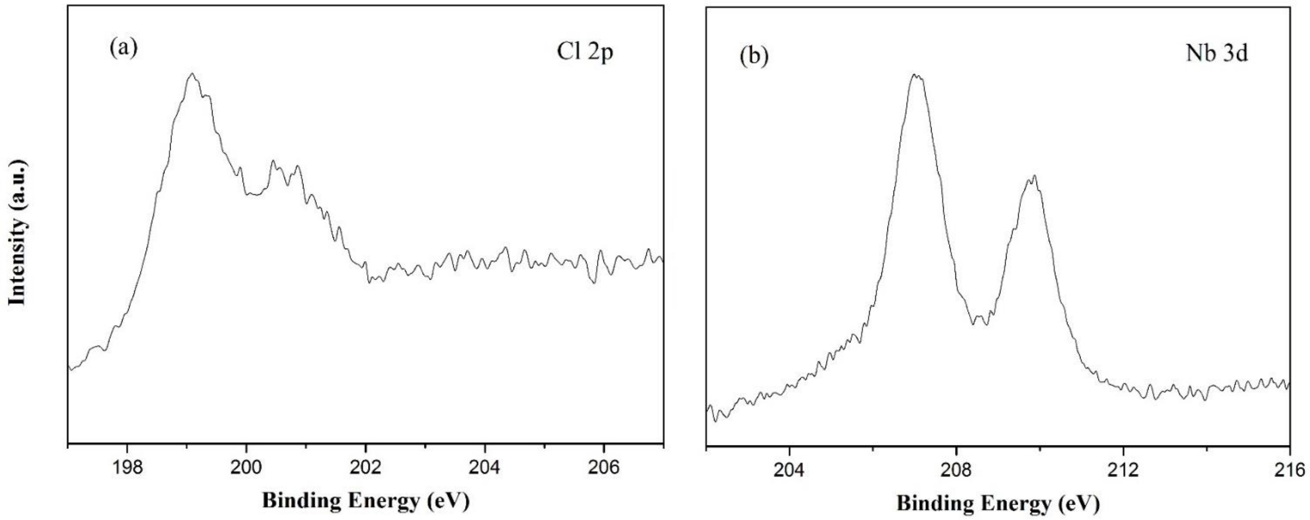

Figure S2 XPS scan spectra of $LiBH_4$-CaH_2 composites with catalysts addition after ball milling (a) Cl 2p in 1mol% $TiCl_3$ doped (b) Nb 3d in 5 wt% NbF_5 doped

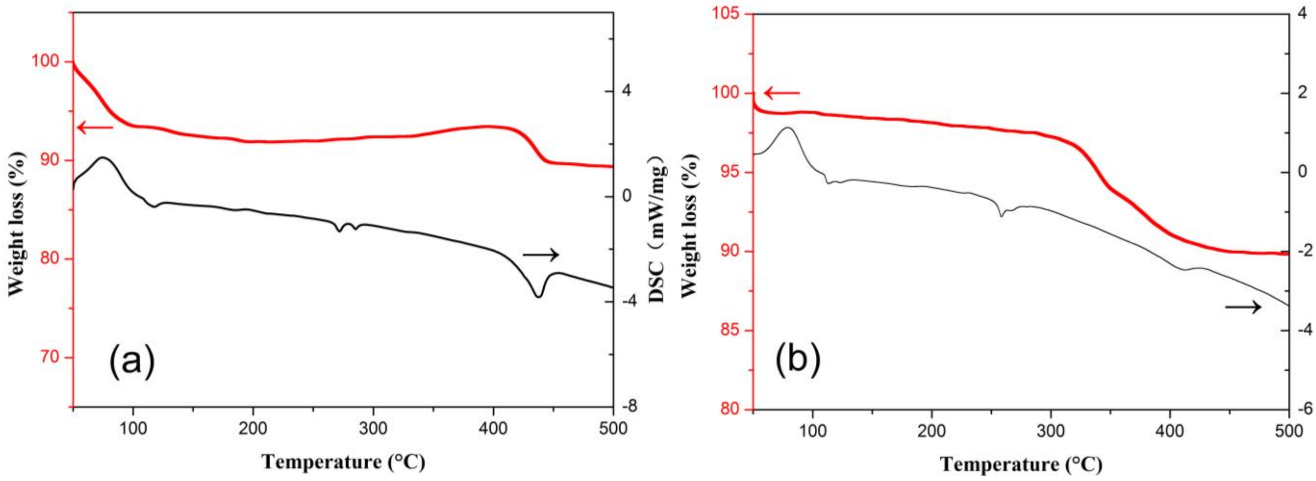

Figure S3 DSC and TGA curves of $LiBH_4$-CaH_2 composites with catalysts additions: (a) 1 mol% $TiCl_3$ doped, (b) 5 wt% NbF_5 doped
